# Turning regenerative technologies into treatment to repair myocardial injuries

**DOI:** 10.1111/jcmm.14630

**Published:** 2019-09-30

**Authors:** Felicia Carotenuto, Laura Teodori, Anna Maria Maccari, Luciano Delbono, Giuseppe Orlando, Paolo Di Nardo

**Affiliations:** ^1^ Centro Interdipartimentale di Medicina Rigenerativa Università di Roma Tor Vergata Rome Italy; ^2^ Dipartimento di Scienze Cliniche e Medicina Traslazionale Università di Roma Tor Vergata Rome Italy; ^3^ Diagnostics and Metrology (FSN‐TECFIS‐DIM) ENEA, C.R. Frascati Rome Italy; ^4^ Wake Forest University School of Medicine Winston Salem NC USA; ^5^ Department of Surgery Wake Forest University School of Medicine Winston Salem NC USA; ^6^ I.M. Sechenov First Moscow State Medical University Moscow Russia

**Keywords:** cardiac cell therapy, cardiac microenvironment, cardiac regeneration, cell‐biomaterial interaction, immunomodulation, tissue engineering

## Abstract

Regenerative therapies including stem cell treatments hold promise to allow curing patients affected by severe cardiac muscle diseases. However, the clinical efficacy of stem cell therapy remains elusive, so far. The two key roadblocks that still need to be overcome are the poor cell engraftment into the injured myocardium and the limited knowledge of the ideal mixture of bioactive factors to be locally delivered for restoring heart function. Thus, therapeutic strategies for cardiac repair are directed to increase the retention and functional integration of transplanted cells in the damaged myocardium or to enhance the endogenous repair mechanisms through cell‐free therapies. In this context, biomaterial‐based technologies and tissue engineering approaches have the potential to dramatically impact cardiac translational medicine. This review intends to offer some consideration on the cell‐based and cell‐free cardiac therapies, their limitations and the possible future developments.

## 1 INTRODUCTION

For decades, the possibility of treating degenerative heart diseases using cell therapy has existed as a vision.^1^, ^2^ Since then, countless studies on the potential of progenitor cells have disclosed unprecedented scenarios about the ability to repair tissue degenerative injuries by substituting dead cells with healthy cells. However, even with an abundance of optimistic data, myocardial repair remains an unmet dilemma. The human adult myocardium displays a limited inherent overhauling capability 3 that poorly ameliorates after hit by an ischaemic insult.^4^, ^5^ Myocardial ischaemic injury results from severe impairment of coronary blood supply inducing irreversible damage in the cardiomyocytes. Consequently, the ischaemic myocardial tissue is permeated by immune cells and myofibroblasts 6, 7 and, ultimately, is sealed by a permanent scar. To circumvent the limitations of the heart's self‐repairing capability, sophisticated long‐term palliative pharmacological treatments are implemented that delay, but do not reverse, the progression of cardiomyocyte death, which inevitably leads to cardiac failure. The treatment for this otherwise incurable condition is a heart transplantation, but, due to the permanent risk of rejection, the shortage of donors, and the high costs, this surgery is often unavailable to patients worldwide. Recent advances in cell biology have provided hope that, to preserve cardiac function, uncontrollable cardiac diseases can be cured by stem cell, or newly generated cardiomyocyte, implantation into the injured myocardium or by enhancing the innate myocardial regenerative programme. However, the paucity of clinically relevant results after years of intense research has dampened the initial hopes. It is likely that new efforts directed to improve the knowledge about stem cell behaviour and their secretome, novel biomaterials and the modulation of the ischaemic environment of the recipient tissue could finally allow for full exploitation of cardiac cell therapy, helping provide great benefit for patients worldwide. With this in mind, the present review intends to offer consideration and to promote the discussion on the cell‐based and cell‐free cardiac therapeutic approaches, their limitations, and their possible future development through biomaterial‐ and tissue‐based engineering technologies.

## 2 IN SEARCH OF THE OPTIMAL CELL TYPE

The aim of cell therapy is to implant functionally healthy cells for irreversibly damaged myocardial tissue to reconstitute the native bioarchitecture, enhancing cardiac function to physiological levels. The first crucial step in this process of repairing injured myocardium is to select the most appropriate cell population(s) to be implanted. A variety of cell types, such as skeletal myoblasts, embryonic stem cells (ESCs), bone marrow‐derived mononuclear cells (BMMNCs), mesenchymal stem (or stromal) cells (MSCs), haematopoietic stem cells (HSCs), endothelial progenitor cells (EPCs), cardiac progenitor cells (CPCs) and induced pluripotent stem cells (iPS), have been isolated and scrutinized as possibilities to repair the damaged myocardium.^8^, ^9^ However, the failure of these cell lines to align with expectations, their genetic instability, the tumorigenic and immunogenic properties, and ethical issues, such as seen with ESCs, has greatly curtailed their potential application in the clinical setting. Prospectively, induced pluripotent stem cells (iPS), obtained by reprogramming patient's somatic cells to exhibit essential characteristics of ESCs cells, hold great promise for heart regeneration, circumventing many hurdles (immune rejection and ethical concerns) that have hampered the extensive use of other cell types. Though iPS technology has potential, it requires further development to decrease the risk of tumour formation 10 and to enhance its capability to maturate as cardiomyocytes (CMs). Presently, only foetal‐like pro‐arrhythmogenic CMs have been generated, and most of the clinical investigation carried out has involved only bone‐derived MSCs and heart‐derived progenitor cells from cardiospheres or c‐Kit + resident cells (kit + CPCs). Other heart‐isolated cell populations such as epicardium‐derived cells, cardiac side population cells, stem cell antigen‐1—Sca‐1 + CPCs,^11^ pericytes 12 and adipose stem cells 13 have also been proposed for cardiac cell therapy. It is important to note that progenitor cells resident to the myocardium have been credited for superior cardiomyogenic potential and higher capability to stimulate cardiac endogenous repair mechanisms. MSCs exhibit limited cardiomyogenic potential and are currently considered for the high secretory profile rather than as exogenous cells to replace lost heart cardiomyocytes.^14^, ^15^ Excellent reviews that were recently published describe the most relevant pre‐clinical and clinical studies of cell‐based therapies.^9^, ^16^

Among the progenitor cells considered for cardiac cell therapy, it is possible that autologous cells will not require immunosuppression. However, mild immunogenicity can be determined via culture, differentiation conditions and epigenetic modifications.^17^, ^18^ Still, several protocols allow for the differentiation of a pluripotent stem cell population in a heterogeneous pool including atrial‐, ventricular‐, nodal‐like cardiomyocytes and immature or undifferentiated cells.^19^ This cell insufficient purity may induce graft‐related arrhythmias when the cells are transplanted into the host myocardium. The potential arrhythmic risk may be attributed to differences in electrophysiological maturity, gap junction alterations, cell orientation and wave propagation between graft and the host myocardium.^20^ Although immature or undifferentiated cells may integrate with host myocardium, they exhibit inadequate electrophysiological properties such as less organized gap junctions that may lead non‐functional electrical coupling with resident cardiomyocytes. One mechanism may involve alterations in the expression of connexin 43 (Cx43), a myocardial gap junction protein that plays an important role in ensuring efficient cell‐to‐cell coupling and the maintenance of cardiac rhythmicity. However, a pro‐arrhythmic substrate into host myocardium can also be sustained by the presence of clusters of uncoupled stem cells that may create an anatomical block inducing the electrical pathway to find a different route.^21^ Bioactive factors, such as hepatocyte growth factor (HGF) and IGF‐1, injected with CPCs into rat infarcted hearts, may increase the expression of connexin 43 in transplanted cells and restore, at last in part, the electrical function in the host myocardium.^22^ However, the progressive evolution of purification and differentiation protocols can improve the electromechanical integration of human pluripotent stem cell (PSC)‐derived CMs with the injured myocardium and, thus, the cardiac function, but a lot remains to be done to suppress the arrhythmic risk in order to obtain clinically reliable cells.^23^, ^24^, ^25^, ^26^

A further major issue concerning the insufficient purity of PSC‐derived CMs is the tendency to form tumours when implanted in vivo. Variegated genetic and epigenetic abnormalities, induced by long‐term in vitro culturing, trigger neoplastic transformation.^27^ On the other hand, a highly purified progenitor cell population is scalable in vitro, helping obtain a sufficient number of cardiomyocytes to repair extensive heart injuries and reduce the possibility of tumorigenesis after transplantation.^25^ To overcome these concerns, several strategies have been proposed to implement differentiation protocols to generate a pure population of CMs.^28^, ^29^ However, it is ambiguous as to how the different cell types, thus far described with stemness characteristics, could contribute to the preservation of the cardiac structure and machinery. Their natural presence in the healthy myocardium indicates participation in the maintenance of the tissue, but, unless they play a specific role in imbricated differentiating steps of a single process, it remains difficult to determine how each different cell population can contribute to the cardiac physiological maintenance and regenerative process.

In adult mammalian hearts, there is a persistent low rate of new cardiac myocyte formation, approximately 0.3%‐1% per year, which, even if greatly enhanced, cannot match the massive demand after ischaemic or non‐ischaemic injuries.^3^, ^30^ This low cardiomyocyte turnover rate meets the demands of active heart cells that cyclically contract every 0.7 second, assuming a heart rate of 70 bpm, for a standard life span of 70‐80 years without apparent fatigue, while experiencing high intraventricular pressures. No material known on Earth can work so intensely without collapsing after a few months or years. This is completed via the participation of different collaborating stem cell populations aggregating to maintain the multicellular bioarchitecture of a mere 250 g of myocardial tissue through a low rate cell turnover. Living organisms are made of several specialized tissues, none of which contain a sole homogeneous cell population. Every tissue is formed by different cell types that are optimally matched with inert components of ECM. The myocardium is considered as a heterogeneous (composite), anisotropic, viscoelastic system made of living and inert materials. This implies that the intravascular or intraventricular injection of a single cell population can hardly meet the complexity of the myocardial bioarchitecture. This is especially true in an environment characterized by altered oxygen tension and high concentrations of humoural and cellular factors, such as after an ischaemic insult. In addition, all cell‐type candidates thus far for cardiac cell therapy display an uncertain differentiating potential in pre‐clinical and clinical trials 8 as well as a poor capability to differentiate into recipient tissue. This is made more difficult because only a small number (<3%) of injected cells engraft into the recipient's myocardium 8, 31 and their contribution to the heart performance remains questionable.^32^

## 3 CELL‐FREE CARDIAC REGENERATION

In most experiments and clinical trials, despite the lack of cellular engraftment and structural repair, there is some degree of functional improvement reported with cell therapy. This suggests that cell therapy is more beneficial in heart failure than in the restoration of the bioarchitecture of ischaemic myocardium.^33^ The beneficial effects, likely, are induced by a complex array of paracrine factors released by engrafted cells that act in a direct fashion or through stimulating angiogenesis, that is oxygen supply, into the myocardium.^34^ The ‘paracrine hypothesis’ is strongly supported by several experiments that observed the beneficial effects induced by stem cell‐conditioned media on infarcted myocardium.^34^, ^35^ Cells secrete several inducible factors that are released into the intercellular space as free‐floating solutes or transported into membranous vesicles collectively termed ‘extracellular vesicles’ (EVs).^36^ This process occurs in physiological conditions as well as during stress and disease.

Extracellular vesicles are categorized into exosomes (ranging from 40 to 100 nm in diameter), microvesicles (ranging from 100 to 1000 nm) and apoptotic bodies (ranging from 1 to 5 μm). Exosomes are small vesicles filled with miRNAs, proteins, lipids and other cellular components. They are released by all cardiac, endothelial and inflammatory cell types, which suggest that they may play an important role in the cardiovascular system, particularly in the ischaemic heart.^37^ The release of cardiac exosomes is enhanced by hypoxia and is a more efficient mechanism of information transfer than free soluble factors. Cardiomyocyte exosomes are decorated with caveolin‐3 and flotillin‐1 on their surface, contain over 1500 different mRNA transcripts and 340 distinct DNA sequences, and display a specific proteomic signature that characterizes their myocardial origin.^36^ Exosome cargo and secretion are modulated by cellular stress and other signals from the tissue microenvironment.^37^ Thus, exosome cargo is not only cell‐type specific, but it also reflects the cell's pathophysiological state as conditioned by the microenvironment. MSCs and CPCs are able to produce exosomes that contain pro‐angiogenic and pro‐survival factors, such as proteins, cytokines and microRNA that stimulate cardiac endogenous repair.^36^ Independent landmark studies reported that exosomes released by human CPCs 38 or human cardiosphere‐derived cells (CDCs) 39 inhibit apoptosis and promote the proliferation of cardiomyocytes, while enhancing angiogenesis. Exosomes from both CDCs and CPCs reduce scar size and improve ventricular function in rodent MI models,^38^, ^39^ the latter being more cardioprotective than bone marrow‐MSC exosomes derived from the same patients, when compared with dermal fibroblast exosomes.^40^ Cardioprotective effects are also induced ‘in vitro’ and ‘in vivo’ by cardiac fibroblast exosomes,^41^ while those released from human normal dermal fibroblasts do not exert these beneficial effects.^38^, ^40^ Finally, BMC and CPCs exosomes increase blood vessel density in the infarct region.^40^ A major role in exosome beneficial effects is played by miRNAs 42; however, how transferred miRNAs are incorporated into an endogenous RISC complex and mediate their effect in competition with large amounts of host miRNA remains to be clarified.^43^ In CPC exosomes, the most represented miRNAs are miR‐146a‐3p, miR‐132 and miR‐210 that display antiapoptotic and pro‐angiogenic properties.^38^ Plasma is particularly rich in exosomes 42 from all tissues that also may elicit cardioprotective effects in cardiac ischaemia.^44^, ^45^ However, owing to comorbidity, the number and cargo composition of plasma exosomes can be highly variable making not yet obvious how this potential can be therapeutically exploited. On the other hand, the different culture protocols used to process cells generate supernatant with non‐comparable exosome compositions that might explain the ambiguous results in cardioprotective experiments and clinical trials.^46^

Exosomes from iPS or ESCs also exhibit cardioprotective functions by reducing apoptosis in cardiomyocytes and cardiac stem cells.^47^ It is important to note that induced pluripotent stem cell (iPS)‐derived extracellular vesicles (EVs) demonstrate safer and more effective cardiac repair than iPS cells in a murine model.^48^ Therefore, the identification of subpopulations of EVs displaying maximal therapeutic potential is of paramount interest for the creation of novel protocols designed to treat degenerative diseases.^49^ This implies that purified secreted factors or vesicles containing factors that are beneficial could be more effective than direct injection of cells in repairing tissue damage. Unfortunately, there is still a gap in knowledge about stem cell secretome and the complexity of its multifactorial action.

Recently, alternative strategies have been developed, instead of implanting stem cells, the turnover of native cardiomyocytes is enhanced by transferring nucleic acids that act intracellularly,^50^, ^51^ or growth factors that act through cell surface receptors 52, 53 or redox regulators.^54^, ^55^, ^56^ The inhibition of the glycogen synthase kinase (GSK)‐3 57 and modulation of the Hippo pathways 58 as well as other molecular mechanisms that induce cardiomyocyte proliferation are also under investigation.^59^ Unfortunately, the strategies that aim to induce endogenous myocyte turnover, although promising, do not guarantee that the resulting contractile cells will be able to counteract heart failure in humans or that a cell neoplastic transformation will not occur. In particular, activation of YAP, the major effector of the Hippo pathway, promotes proliferation and regeneration of cardiomyocytes after myocardial infarction,^60^ but it can bear notable risks in promoting cancer.^58^ In addition, the induction of cardiomyocyte turnover alone could not be sufficient to repair the complex myocardial architecture.

**Figure 1:**
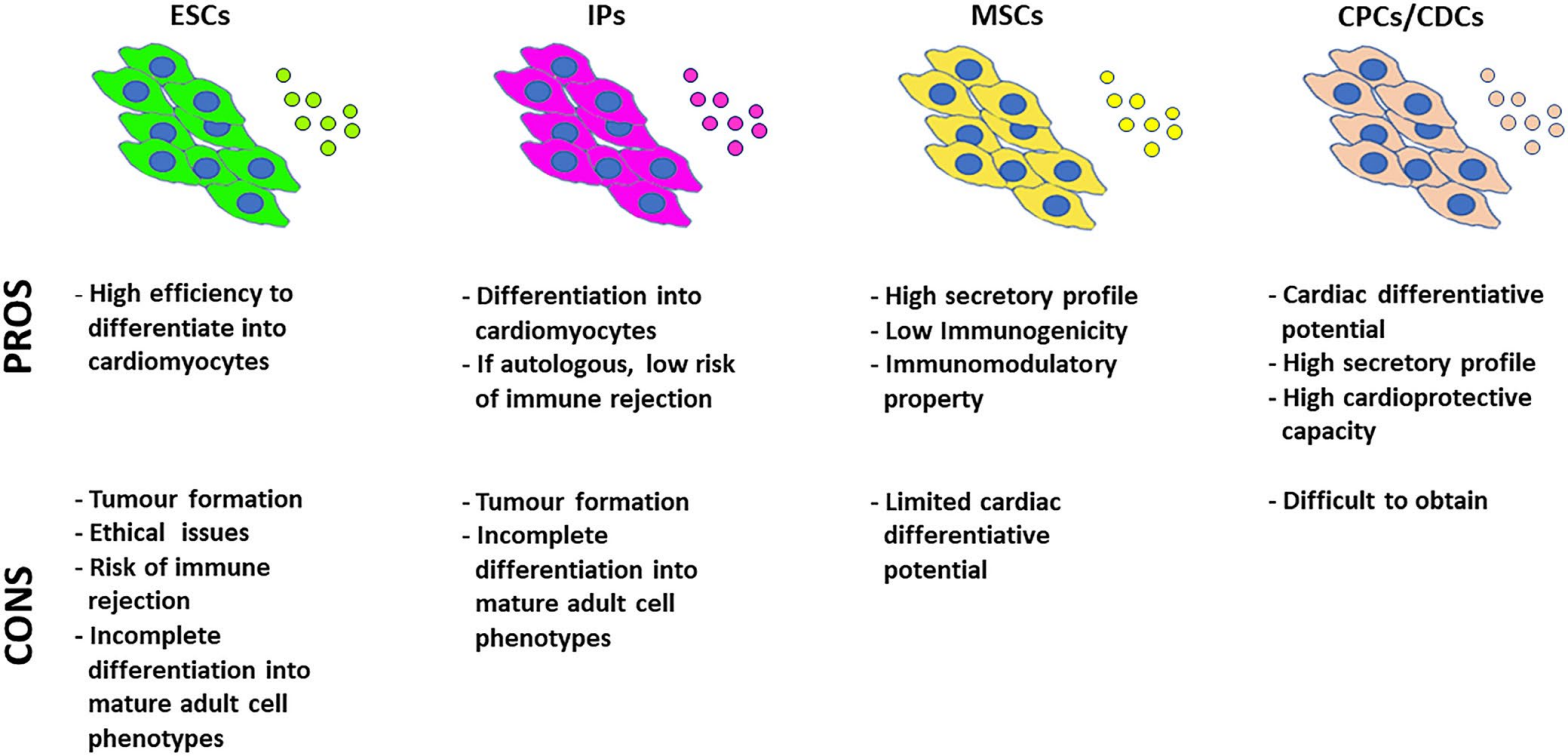
The main properties of candidate cells for cardiac repair. The stem/progenitor cell sources most often utilized in cardiac regenerative applications and their advantages and disadvantages

Altogether, there are two major hypothetical mechanisms to promote cardiac repair by transplanted cells. The first implies a direct differentiation of stem/progenitor cells in cardiomyocytes or vascular cells to replace the lost myocardium. The second implies indirect stimulation of the cardiac tissue regeneration through paracrine factors released by the cells. The specific therapeutic goal influences the selection of optimal cell type. The main properties of candidate cells for cardiac repair are shown in the Figure 1.

## 4 SUPPLYING CELLS WITH PROPER MICROENVIRONMENTAL CUES

Many uncertainties must be addressed regarding both cell‐based protocols and approaches that harness myocardial endogenous repair mechanisms. If the primary objective is to regenerate the damaged myocardium with exogenous cells, it is evident that restoration of an appropriate cell‐ECM crosstalk could lead to clinically relevant outcome. In this context, an irremissible step in establishing efficient and safe protocols for cell therapy is to develop novel technologies that preserve stem cell capacity outside of its native tissue. The design of these technologies likely entails complex methods, rather than physical devices, to deliver important signals released in the native stem cell ecosystem, allowing cells damaged by isolation to recover in a proper microenvironment.

Current protocols call for progenitor cells to be isolated from their native microenvironment and cultured in vitro, which fails to simulate the complexity of the physiological cardiac cellular environment. After a rough isolation procedure, the complex array of signals, that control stem cells in their microenvironment, is missing and is replaced by meagre signals from conventional experimental culture conditions. The final goal of this process is to over‐expand the cell culture to obtain adequate cells to repair the injured tissue. Unfortunately, the over‐expansion of stem cells in vitro shortens their lifespan and their nuclear DNA tends to be unstable during long‐term culture. It is plausible that the pattern of growth factors and cytokines released by cultured cells vs. native stem cells is substantially different, and highly variable depending on culture conditions.^35^, ^61^ Hence, after days in long‐term culture conditions, cells suffer further disruption, such as enzymatic detachment, suspension in a physiological solution and exposure to high pressure upon injection into resilient contracting tissue, which lessens their attachment and regenerative ability. As a result, cultured stem cells do not retain their original characteristics and, thus, become unfit for successful implantation. The most crucial step in this procedure to implant efficient cells into the myocardium is to secure isolated stem cells during their ex vivo transit. This can be accomplished by developing a system or ‘cradle’ that emulates, in vitro, the native ECM’s ability to release physical and biochemical signals to these isolated cells while transferring them from native to recipient tissue.

The ‘cradle’ is intended to allow stem cell structural and functional recovery after traumatic isolation, and to ease transition into a foreign microenvironment before implantation. Its major characteristics should include the following: (a) an environment that is intrinsically non‐cytotoxic, non‐immunogenic and minimally pro‐inflammatory; (b) a design that addresses tissue bioarchitecture as well as the shape and size; (c) an architecture that optimizes cell, nutrient, gas, and biomolecule transport, and facilitates blood vessel and nerve development; (d) a material that dynamically adapts to the ever‐changing cell microenvironment; (e) optimal surface or interface energy characteristics that facilitate cell adhesion and function; (f) physical cues, such as stiffness and topology, that release mechanical signals, promoting cell differentiation and architectural organization; and (g) an orchestration of molecular signalling via delivery of biological molecules.

**Figure 2:**
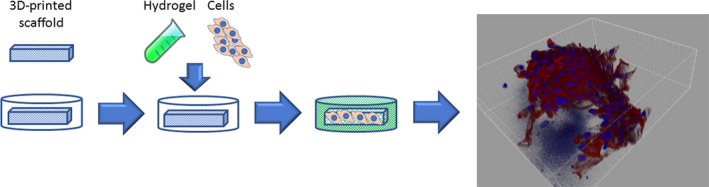
3D scaffold‐in‐scaffold mimicking the ECM organization. A stiffer woodpile microstructure has been embedded in a biocompatible hydrogel in which the cardiac progenitor cells were incorporated. After a long‐term culture (3 wk), mechano‐structural signals from 3D structure induced cells to commit towards the cardiomyocyte phenotype as shown in the 3D rendering confocal image (panel right) in which α‐sarcomeric actinin expression is displayed in red and nuclei are stained by DAPI (blue). The image comes from a set of experiments made in our laboratory. The essential results were published 65

Several experiments indicate that hydrogels, formed by a three‐dimensional network of hydrophilic polymer chains surrounded by a water‐rich environment,^62^ are a promising material for the cradle infrastructure. Cells dynamically tune their inner tensile state through actomyosin contractility and organization of the F‐actin cytoskeleton in response to physical stimuli in the microenvironment, which is sensed through integrins and other adhesive proteins.^63^ Physical and mechanical contact between cells and their extracellular matrix as well as soluble signals and metabolic pathways are highly integrated by the mechanosensitive transcriptional regulators YAP and TAZ to control multiple aspects of cell behaviour, including proliferation, cell plasticity and stemness.^64^ Three‐dimensional hydrogels optimally mimic the native ECM architecture, releasing physical signals that can be tuned and transduced through integrins and other adhesion molecules, modulating intracellular tensile state. Monitoring YAP and TAZ activity, in cells grown on hydrogels and other material‐based platforms with defined chemical, mechanical and physical parameters, leads to a deeper understanding of how mechanical cues can influence individual cell behaviour. This approach guides the rational design of novel classes of biomaterials that are specifically tailored for determining specific intracellular effects that address cell fate and tissue bioarchitecture.^64^ In this context, a promising example of the cradle infrastructure is represented by the ‘scaffold‐in‐scaffold’ design, in which a stiffer woodpile microstructure is embedded in a biocompatible hydrogel and the whole structure is soaked into the culture medium. Stem cells are incorporated into the hydrogel, successfully grown and induced to differentiate into cardiomyocytes on the woodpile structure that rudimentarily mimics the organization of the ECM.^65^ In the ‘scaffold‐in‐scaffold’ design, a three‐dimensional intricate network of fibres dynamically releases mechano‐structural signals into the cells (Figure 2). Natural and synthetic materials are under investigation for use in this novel family of structures, but much remains to be examined about their optimal chemistry and design, and, above all, the adaptive strategies utilized by cells when in the presence of unfamiliar materials.

In native tissues, cells and ECM are in a state of ‘dynamic reciprocity’. Signals from the ECM modulate cell behaviour, and cells, in turn, modify the organization and composition of the ECM.^66^ In this context, cardiac differentiation is propelled by a complex dynamics of biochemical, biophysical and bioelectrical signals.^67^ The literature indicates that different matrix microarchitectures and surface topographies can have drastic implications for the behaviour of cardiac precursor cells, influencing their differentiation and contractile capacity. Therefore, the cradle, used for transferring cells for implantation into a damaged myocardium, must provide a variety of physical and biochemical signals to emulate the original niche environment, which helps preserve as much of the original capability of these progenitor cells as possible. These optimally maintained progenitor cells could be used to manufacture in vitro tissue strips that could subsequently be implanted into the damaged myocardium, improving cell retention at the infarcted site and after‐implant cell viability. In this respect, three strategies are still under scrutiny:

### 4.1 Cell sheet technology

This process takes advantage of a cell's natural ability to secrete and assemble matrix components in culture, allowing the manufacture of cell sheets on thermo‐sensitive materials. These sheets can be easily detached and further assembled into thicker multilayer tissues.^68^ The technology allows for the fabrication of ex vivo engineered cardiac tissue that could be directly implanted for the delivery of human cardiac progenitor cells into the myocardium.^69^

### 4.2 Organ decellularization

This technology uses the innate extracellular matrix (ECM) of multicellular organisms as a template for organ fabrication. The rationale for doing so is that the role of the ECM goes far beyond mechanical support and architecture and includes—as previously illustrated 70—effects on molecular composition, cell adhesion, signalling and binding of growth factors. In addition, its mechanical characteristics (eg, stiffness and deformability) contribute to the determination, differentiation, proliferation, survival, polarity, migration and behaviour of cells. The ECM template is obtained through the decellularization—that is the clearance of the cellular compartment—of the tissue organ of interest, in this case the heart.^70^, ^71^ Recent studies have showed that decellularized human hearts can be repopulated with cardiomyocytes derived from human IPS cells.^72^ These cardiomyocytes successfully grafted onto cardiac scaffolds and showed sarcomeric structure and electrical conductivity like functionally beating myocardial tissue. Although this research provides the foundation to generate therapeutic grafts on a human scale, several issues remain, such as the homogeneity of different preparations, the possible transfer of viruses and potential rejection.^73^

### 4.3 Scaffold technology

This method's goal is to provide a biocompatible polymeric scaffold that mechanically supports 3D cell growth and differentiation, allowing formation of new tissue for implantation. Numerous polymeric materials and scaffold designs have been scrutinized, including hydrogels. In general, materials of natural or synthetic origin are used alone or with embedded bioactive molecules. Encouraging results have also been obtained from combining different materials to obtain a composite scaffold.^74^ Inert materials interact with cells to help determine their fate.^75^, ^76^ Three‐dimensional (3D) biomaterial constructs, resembling the physico‐chemical properties, micro‐topography and mechanical characteristics of the myocardium, can induce non‐resident and resident stem cells to initial cardiac commitment even in the absence of further stimuli.^77^ An important aspect is the biomaterial microstructure geometry which can drive the CPC alignment and induce cardiomyocyte commitment.^67^ However, current scaffold designs have been redirected to manufacture templates that are able to deliver the following: (a) physical signals, which are released to the cells through appropriate stiffness (approximately 25‐35 kPa) and texture (pores that allow the flow of oxygen and nutrients, and the remove waste) and (b) biological signals, which are transported by free‐floating molecules of morphogens, growth factors and microvesicles, charged with proteins and nucleic acids sequences, that are able to stimulate cells. Paracrine substances from stem cells and therapeutic agents can be incorporated into biodegradable polymers to form drug‐releasing systems. Recent literature showed that synthetic microparticles coated with human stem cell membranes lead to the preservation of viable myocardium and augmentation of cardiac function in rodent models with MI.^78^ Similar microstructures loaded with natural bioactive compounds that generate a broad biological effect, such as linolenic acid, have shown a strong protective effect on cardiac cells.^79^, ^80^ The cradle infrastructure can also be engraved with subsystems that release biological signals on‐demand.^81^ Different families of exosomes or vesicles containing biologically active substances 82 can cause a specific profile of release for the support of different phases of cell development. However, the generation of thick vascularized tissues still remains an unmet challenge in cardiac tissue engineering. Large human cardiac muscle patches of clinically relevant dimensions (4 cm × 2 cm × 1.25 mm) generated by suspending a mixture of three cell types in a fibrin matrix have shown their capability to significantly improve the recovery from heart attack injury in large animals. Interestingly, iPS cells were reprogrammed from cardiac‐lineage cells rather than from dermal fibroblasts and induced to differentiate in cardiomyocytes, smooth muscle cells and endothelial cells.^83^

A further leap forward is represented by the extensive use of 3D printing technology that could allow the realization of more efficient contractile tissue and more precise print of small‐diameter blood vessels. A 3D‐printed thick and vascularized human cardiac tissue manufactured using patient's own cells and biological materials has shown huge potential as future approach based on the use of engineered personalized tissues for organ replacement, even if the printed blood vessel network is still limited.^84^

This abundance of research has generated novel and sophisticated designs for template‐releasing signals, but their complexity is currently inferior to the original cardiac texture and function, making difficult to translate promising experimental results into clinical practice.

## 5 A STABLE BUILDING RESTS ON FIRM FOUNDATIONS

The possibility that new cells thrive in damaged myocardium is improved if the administered cells are adequately purified and qualitatively optimal, and if the recipient injured tissue is under controlled conditions. In fact, a significant obstacle for cardiac regenerative medicine is the hostile microenvironment of the infarcted tissue that limits the engrafted cell's ability to survive and repair the myocardium. The fate of transplanted cells is heavily affected by ischaemic myocardium because it is deprived of blood supply and turmoiled by necrotic, apoptotic, and non‐resident cells, by increased collagen density, and by an intricate array of non‐beneficial soluble factors. The physical signals related to the stiffness of the recipient myocardial tissue are modified by the presence of debris, oedema and fibrosis, while the tissue structure is dwarfed by the misalignment of cells, and the altered vascular bed. At the same time, chemical signals, such as tissue pH, metabolites and intracellular biomolecules, which are dispersed in the extracellular space, are deeply influenced by the amount of apoptosis, necrosis and bioarchitectural devastation caused by ischaemia. Due to tissue damage and necrosis of cardiac cells, ‘danger signals’ such as extracellular matrix (ECM) breakdown products and mitochondrial DNA activate innate immune pathways and trigger an intense inflammatory response,^85^ while non‐myocardial cells invade the injured tissue, creating a completely new ecosystem in which foreign cells struggle to survive. Initially, the immune response activates neutrophils that secrete proteases, which promote cardiac remodelling, and chemokines that enhance the recruitment of monocytes into the infarcted area. Monocytes transdifferentiate into macrophages and, together with neutrophils, release pro‐inflammatory cytokines, such as TNF, interleukin 1 β (IL‐1β) and interleukin 6 (IL‐6) that are detrimental to surviving cardiomyocytes. This inflammatory response is necessary to remove cellular debris. At the same time, the inflammatory microenvironment created by the infiltrated neutrophils and monocyte‐derived macrophages is essential for cardiac repair.^86^ Macrophages play critical roles in tissue repair after injury.^87^ Indeed, infarct macrophages exhibit a pro‐inflammatory M1 phenotype early and become polarized towards an anti‐inflammatory M2 phenotype later post‐MI. These M2 macrophages are essential for inducing infarct healing, because the depletion of M2 cardiac macrophages drastically impairs healing and the reparative ability of progenitor cells, worsening the disease outcome.^88^, ^89^ In addition, factors released from macrophages and others present in the MI environment, such as stromal cell‐derived factor 1 (SDF‐1α), may recruit and activate exogenous or resident progenitor cells 90, 91 which in turn can affect macrophage polarization and other immune cells.^92^ Hence, crosstalk between progenitor cells and immune cells could play a pivotal role in the cardiac repair.

However, the inflammatory cascade also stimulates fibroblast proliferation and transdifferentiation in myofibroblasts that produce excessive extracellular matrix, mainly composed of collagens, resulting in the formation of scar tissue. Initially, this scar tissue replaces lost cardiomyocytes and prevents rupturing of the ventricular wall. The progression of matrix deposition from activated myofibroblasts leads to increased ventricular stiffness and impairs contraction.^93^ The role of fibroblasts may extend beyond their contribution to scar formation and cardiac matrix remodelling. Fibroblasts, previously thought to act as simple electrical insulators, can be electrically interconnected among themselves and to other cells, including cardiomyocytes. In addition, fibroblasts are noted to perform important autocrine and paracrine signalling functions due to their abundance, strategic location, phenotypic plasticity, ability to communicate with different cell types and active participation in cardiac mechanical and electrical activity.^94^, ^95^ Thus, cardiac fibroblasts may be key therapeutic targets in cardiac remodelling and repair. Studies in models of cardiac regeneration have shed light on further aspects of cardiac fibroblast biology. In zebrafish, the genetic ablation of fibroblasts impaired cardiomyocyte proliferation, while their inactivation led to fibrosis regression and cardiac regeneration.^96^ These data suggest that fibroblasts could be key players in the cardiac regenerative process and scar resolution. Thus, the possibility of modulating fibroblast activation could represent a promising therapeutic strategy to improve the MI outcome.

Recent studies show that mature scars are made of dynamic, metabolically active tissue that is able to characterize the fate of experimentally implanted cells, likely, through the inflammatory reaction induced by the cells themselves.^94^ The abundantly present ECM is interlaced with phenotypically diverse cells, such as interstitial fibroblast‐like cells that are both functionally and structurally heterogeneous, endothelial cells, vascular smooth muscles, surviving cardiomyocytes, neurons, adipocytes and immune cells.^94^

In post‐ischaemic myocardium, immune system cells play a vital role in co‐ordinating cardiomyocyte and non‐cardiomyocyte responses during maladaptive remodelling. They clear up the debris, initiate the wound healing process and form proper scar tissue.^97^ During cardiac injury, macrophages not only drive a robust inflammatory response and matrix remodelling, but they are required for the resolution of inflammatory and reparative activities, including angiogenesis and myocardial proliferation.^98^ However, the balance between inflammatory and reparative phases is delicate and necessitates proper equilibrium to prevent excessive inflammation, adverse remodelling, or inadequate stimulation of repair. Excessive immune response can actually interfere with repair or exacerbate damage.^99^

Transplanted cells can modulate the immune system. During the last decade, MSCs have been suggested as potent modifiers of the immune system with the ability to shift the balance towards the reparative phase and reduce the inflammatory process.^100^ Moreover, in contrast to other stem/precursor cell types, MSCs can evade immune system detection, due to the absence of expression in the MHC class II molecules, with their potential applications extending towards allogenic settings.^101^ In the presence of inflammatory cytokines, such as tumour necrosis factor‐α, and interleukin‐1β,^99^ MSCs are primed to release soluble factors that counteract the activation, proliferation and maturation of cells that carry out both adaptive and innate immunity.^102^ MSCs affect every immune cell to a certain degree and impair T‐cell proliferation and differentiation, cytokine secretion, and cytotoxic potential.^103^, ^104^ MSCs suppress the formation of TH1 and TH17 lymphocytes, which are essential for the activation of cytotoxic T cells and the enhancement of phagocytic capacity of neutrophils and macrophages. Meanwhile, MSCs enhance the formation of TH2 lymphocytes, which have a more immunotolerant phenotype and produce anti‐inflammatory cytokines, such as IL‐4 and IL‐10.^103^ In addition, MSCs suppress neutrophils, dendritic cells and natural killer (NK) cells,^105^, ^106^ while induce the conversion of T cells into T‐regulatory cells,^107^, ^108^ which have cardioprotective and regenerative effects.^109^, ^110^ MSCs also enhance macrophage differentiation into the M2 subtype, which reduce pro‐inflammatory cytokine production, and stimulate cardiac reparative pathways, anti‐inflammatory mediators and angiogenesis.^103^ Recent evidence shows that MSC modulation of inflammation and immune response after myocardial infarction could be operated through exosome release, influencing myocardial repair and remodelling.^100^, ^111^ MSC‐derived exosomes may reduce cardiac inflammation after myocardial injury modifying the polarization of M1 macrophages to M2 macrophages via shuttling miR‐182.^112^ In addition, exosomes from MSCs are able to decrease neutrophil infiltration 113 and T‐cell proliferation 114 enhancing cardiac repair. CPC‐derived exosomes display similar capacity for ‘in vitro’ strong immunosuppression of the T cells, when compared with MSC‐derived exosomes.^115^ CDC‐derived exosomes can also exert immunomodulatory effects reducing leucocyte infiltration 116 and promoting macrophage polarization towards an M2 anti‐inflammatory phenotype 117 after myocardial injury. All these studies show that exosomes reassemble the immunomodulatory effects of the cells from which they derive. Thus, the exosomes could be used as immunomodulating agents in the myocardial environment.

A promising therapeutic approach could be the modulation of the harsh post‐ischaemic myocardial environment to create a more suitable ‘foundation’ that allows engrafted cells to grow, differentiate and integrate with the recipient surrounding tissue. This could be achieved by associating novel pharmacological strategies to the transplantation of MSC‐derived exosomes before administering precursor cells that are prone to cardiac differentiation.^118^ In this respect, it is crucial to define when preserved progenitor cells should be administered to injured myocardial tissue. The final step of the post‐ischaemic process, when fibrosis ultimately claims the injury, is too late. It is more plausible to administer stem cells during the initial (humoural) or intermediate (cellular) stage of the post‐ischaemic process to timely suppress and spatially contain the post‐infarction inflammatory reaction. Consistently, preliminary data from clinical trials have shown that a modest, but significant, improvement in left ventricular function is achieved when cell therapy is administered 4‐7 days after the myocardial injury occurred.^119^ The mechanisms underlying the efficacy of cell therapy based on timing require clarification before it is considered clinically.

Infarction pathophysiology is very heterogeneous. Pro‐inflammatory signalling can be prolonged with associated dilatative remodelling and systolic dysfunction, while other patient subpopulations exhibit a marked hypertrophic and fibrotic response associated with diastolic dysfunction. The stringent categorization of different infarcted subpopulations is preliminary to the strategy that will be applied in preparation of the injured myocardium to accept exogenous cells and allow their differentiation. In this regard, it must be considered that inflammatory factors are pleiotropic. They not only negatively affect the integrity of the recipient tissue, but they also promote regeneration. The challenge will be to design highly selective treatments that can either enhance or repress those effects that could be advantageous to repair injured myocardium.

## 6 STEM CELL VULNERABILITY REQUIRES A SMOOTH DELIVERY

A successful cardiac regenerative therapy also depends on cell delivery routes and procedures. Cells can be administered by intravascular (intravenous or intracoronary) or intramyocardial injections. Intramyocardial cell injections allow the direct delivery of cells into the infarcted area and, thus, it is the most precise type of delivery.^120^, ^121^ However, the intramyocardial injection of progenitor cells usually requires open‐chest procedures increasing the risk of secondary injuries and infections in MI patients. The intravenous administration is the least invasive and clinically convenient way, but its efficacy is undermined by the entrapment of most of the delivered cells in the pulmonary capillaries with consequent low retention in the myocardium infarcted area. Cell infusion through percutaneous coronary catheters usually results in better cell engraftment,^121^ when compared with systemic delivery, such as intravenous injection. However, it remains to be defined whether it would be optimal to deliver the new cells in the infarcted area or in surrounding region. Indeed, the challenge for all intravascular approaches is how to precisely target cells so that the therapeutic agent (ie, drugs, cells, cell‐derived exosomes, vesicles, micro‐ or nanoparticles) can interact with the heart's infarcted area and impart their therapeutic benefits.^122^ Of primary interest is, therefore, the development of better methods to enhance the targeting ability of cells or other therapeutic agents, such as exosomes, for injured heart treatment. Intravenously injected exosomes are predominantly entrapped into the liver,^123^ while their intramyocardial delivery is more effective than intravenous and intracoronary injection.^116^ In order to facilitate minimally invasive implantation procedures, different strategies are under scrutiny to target cells or exosomes to the injured heart. Among others, exosome surface may be modified to improve targeting to cells of interest.^124^ Exosome‐producing cells can be molecularly engineered to enrich the presence of specific molecules on the exosome surface in order to target damaged myocardium. Exosomes derived from CPCs engineered to overexpress CXCR4, a receptor of the chemokine stromal cell‐derived factor 1 (SDF‐1), increase myocardium homing and cardioprotective effects in a rat model of ischaemia/reperfusion injury after systemic delivery.^125^ The enrichment in ligands can also be achieved by directly conjugating a synthetic myocardium targeting peptide, such as the cardiac homing peptide (CHP), to the surface of intravenously infused CSC‐derived exosomes.^126^ In addition, cells labelled with superparamagnetic nanoparticle can be guided to the injury site by an external magnetic field placed above the heart during the injection enhancing the retention/engraftment in the damaged area and multiplying the therapeutic benefit.^122^ The magnetic targeting strategy is non‐toxic and can be applied universally to many cell types and exosomes.^127^ All these targeting strategies dramatically increase the retention of therapeutic agents, such as cells or exosomes, even after intravenous delivery.

Catheter‐based applications to delivery cells and polymerizable hydrogels may significantly enhance cell retention.^128^ However, for injectable hydrogels, catheter technology needs to be further developed to preserve the liquid pre‐polymer during catheter transit to the injection site, while allowing fast pH‐ or temperature‐dependent polymerization, when released into the infarcted site.^121^

Patches or scaffolds are so far delivered to the epicardium via open‐chest surgery.^69^ Future efforts should focus on developing less invasive systems based on next generations of ‘smart’ biomaterials that could facilitate cell and bioactive factors delivery. Scaffolds made of injectable shape‐memory biomaterials may allow non‐invasive implantation surgery based on the use of small catheters. Indeed, cardiac patches performed with these flexible materials may significantly improve cardiac function and are promising for clinical translation.^129^

## 7 CONCLUSIONS

**Figure 3:**
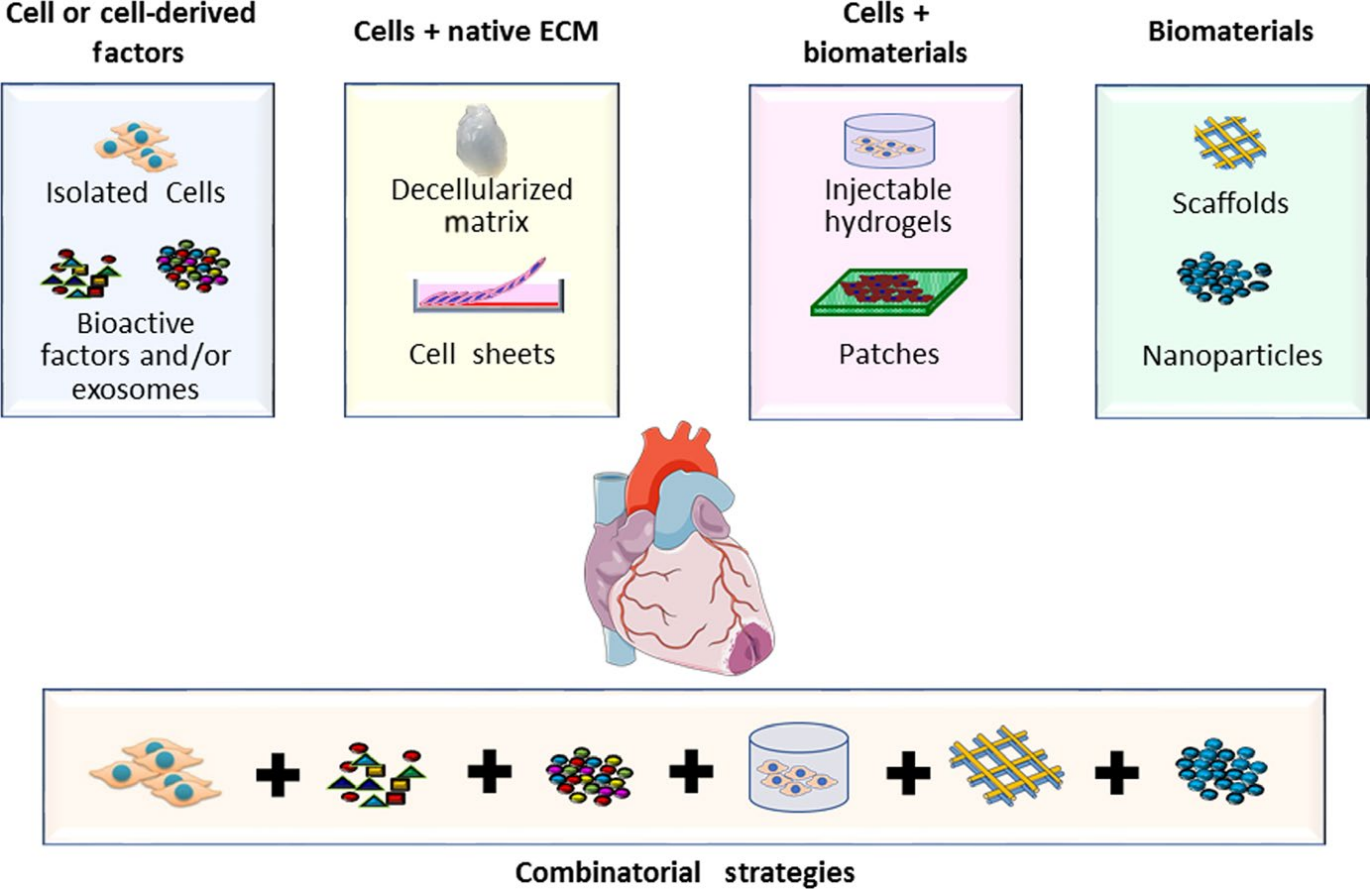
Evolution of regenerative strategies for heart diseases. The first era of cardiac regenerative medicine has been focused on the exclusive use of strategies based on cells (multiple types of stem/progenitor‐like cells) and/or paracrine factors. Subsequently, strategies aimed at restoring the appropriate cell/ECM crosstalk through cell growth on natural or synthetic ECM were developed. Biomaterials may be used to support cells and extracellular signals release. Next‐generation therapies for cardiac repair are directed towards combinatorial approaches. Adult heart in figure was created using Servier Medical Art

Altogether, it is apparent that cardiac cell therapy, in any form, is still in its primary stages. No consolidated, standardized strategies or gains in the field are available, but a constellation of basic, pre‐clinical and clinical results whose correlation is not yet systemically understood. A unified effort is crucial (a) to improve the knowledge about the mechanistic principles presiding embryonic development and (b) to optimize the current and future knowledge about stem cells to create standardized, safe and efficient protocols that can be ultimately applied in a clinical setting. Among others, cell stemness should be defined via novel markers of structural components or specific arrangements of the secretome. It should also be characterized by considering their adaptive behaviour when seeded on a predetermined arrangement of differently designed biocompatible surfaces. Finally, the challenge of complete myocardial regeneration will require (a) the improvement of the recipient myocardial tissue environment before the implantation of therapeutic agents (cells, exosomes, etc), (b) the development of novel biomaterial‐based technologies and combinatorial approaches, and (c) the refinement of the delivery protocols (Figure 3). These approaches, although not yet ready for clinical practice, will be vital to enhance current understanding of the mechanisms underlying the cardiac reparative processes at the molecular, cellular and tissue levels. In addition, these approaches will lead to the discovery of more refined pharmacological therapies that favour clinical applications of cell treatment.

## CONFLICT OF INTEREST

The authors declare no conflict of interest.

## AUTHOR’S CONTRIBUTION

FC, LT and PDN conceptualized and designed the manuscript. AMM and LD contributed to the analysis of the literature. LT and GO contributed to the critical revision of article. FC and PDN wrote and supervised the manuscript. All authors read and approved the final manuscript.

## Data Availability

Data sharing is not applicable to this article as no new data were created or analysed in this study.
